# Accuracy of next-generation sequencing for rapid diagnosis of tuberculous pleurisy: A protocol of systematic review and meta-analysis

**DOI:** 10.1371/journal.pone.0319175

**Published:** 2025-02-25

**Authors:** Yuyang Ling, Yanqin Shen, Xudong Xu, Guocan Yu

**Affiliations:** 1 The Second School of Clinical Medicine, Zhejiang Chinese Medical University, Hangzhou, Zhejiang, China; 2 Department of Nursing, Hangzhou Red Cross Hospital, Hangzhou, Zhejiang, China; 3 Zhejiang Tuberculosis Diagnosis and Treatment Center, Hangzhou Red Cross Hospital, Hangzhou, Zhejiang, China; University of Szeged Institute of Biology: Szegedi Tudomanyegyetem Biologia Intezet, HUNGARY

## Abstract

**Background:**

Early, rapid and precise diagnosis of tuberculous pleurisy is difficult. Next-generation sequencing (NGS) has been used for the diagnosis of TB in some high-income countries. We completed the protocol of the study in order to better guide the systematic review and meta-analysis for evaluating the role of NGS in the diagnosis of tuberculous pleurisy, which was the purpose of the current study.

**Methods:**

We designed and drafted this protocol as per the Preferred reporting items for systematic review and meta-analysis protocols (PRISMA-P) guidelines. Three English databases (Cochrane Library, Embase and Medline via PubMed) and two Chinese databases (China National Knowledge Infrastructure and Wanfang Database) will be used to search for relevant studies. We followed the PICT principles to develop the eligibility criteria. Once the final compliant literature is identified, we will extract the valid information from the literature for statistical analysis. We will assess the quality of the included studies using the Quality Assessment of Diagnostic Accuracy Studies (QUADAS-2) tool. When there are more than 4 eligible studies, we will use Stata (version 15.0; Stata Corp., College Station, TX, the USA) with *midas* module to conduct the relevant meta-analysis and plot the relevant forest plots and curves. When there are less than 4 eligible studies, we will use Meta-DiSc (version 1.4) to perform the associated meta-analysis and plot the associated forest plots and curves. We will use the *I*^2^ statistic to assess heterogeneity between included studies. We will conduct meta-regression analysis, subgroup analysis and sensitivity analysis to explore possible sources of heterogeneity when significant heterogeneity exists.

**Systematic review registration:**

PROSPERO Registration number: CRD42024568791

## Introduction

Infection with *Mycobacterium tuberculosis* (MTB) in some humans can lead to tuberculosis (TB), especially the people once exacerbated by risk factors, like HIV, malnutrition, smoking and diabetes. TB is an ancient infectious disease that poses a serious threat to public health [1]. TB can be broadly categorized into pulmonary TB and extrapulmonary TB (EPTB) based on the site of infection [2]. EPTB includes MTB infections in all organs except the lungs. Pulmonary TB involvement of the pleura or MTB infection of the pleural cavity can lead to tuberculous pleurisy, which is a common type of EPTB [3]. The main manifestation of MTB infection in the pleural cavity is pleural effusion, and due to the confined nature of the pleural cavity, thoracentesis is usually required to obtain a pleural effusion specimen [3]. The altered nature of tuberculous pleural effusion is not specific, and at the same time, the level of pathogenic bacteria in the effusion is extremely low, making early, rapid and precise diagnosis of tuberculous pleurisy difficult [4]. Misdiagnosis or delayed diagnosis can easily occur, delaying appropriate treatment and leading to the development of complications such as pyothorax formation and thoracic constriction, affecting the patient’s prognosis [5].

The role of acid-fast bacilli (AFB) smear and culture, the traditional TB tests, in tuberculous pleurisy is low [6]. AFB smear is quick and easy to perform, although its specificity in pleural effusion was high (97.6%), the sensitivity was low at 13.9% and was not a valid test [7]. AFB culture in pleural effusions is also unsatisfactory (sensitivity was only 27.5% and specificity was 100%) and it takes weeks to get results, which makes it unsuitable for early and rapid diagnosis [8]. Molecular biology tests, which are currently in full swing, have also hit a brick wall in the diagnosis of tuberculous pleurisy, and their diagnostic accuracy is not as good as it is in other types of TB. As an analogy, a meta-analysis showed that the sensitivity of Xpert MTB/RIF for diagnosing tuberculous pleurisy was only 11%-34%, while specificity was 99%-100% [9].

Next-generation sequencing (NGS) has been used for the diagnosis of infectious diseases in some high-income countries, including TB, where NGS has demonstrated diagnostic accuracy superior to other tests [10]. A meta-analysis showed that the combined sensitivity and specificity of NGS for detecting MTB was 67% and 100%, respectively [11]. NGS may be a very good tool in the diagnosis of tuberculous pleurisy. However, the diagnostic value of NGS in tuberculous pleurisy has not yet been comprehensively evaluated and analyzed, and in order to systematically evaluate the role of NGS in the diagnosis of tuberculous pleurisy, we propose to conduct a systematic review and meta-analysis. Before conducting this systematic review and meta-analysis, we completed the protocol of the study in order to better guide the meta-analysis, which was the purpose of the current study.

## Methods

### Design and registration

In order to standardize the systematic review and meta-analysis, we first developed this study protocol and registered it on the PROSPERO platform (CRD42024568791). This protocol was developed based on public databases and did not require ethical review. We designed and drafted this protocol as per the Preferred reporting items for systematic review and meta-analysis protocols (PRISMA-P) guidelines [12]. We will report the results according to the Preferred Reporting Items for a Systematic Review and Meta-analysis of Diagnostic Test Accuracy Studies: The PRISMA-DTA Statement when the final meta-analysis is completed [13].

### Eligibility criteria

We followed the PICT principles to develop the eligibility criteria [14]. P (participants): Participants with suspected tuberculous pleurisy will be included; age, gender, and race will not affect patient inclusion. I (index test): Index test in this study protocol refers to NGS. C (comparative test): For diagnostic test accuracy (DTA) study, the comparative test is the reference gold standard. Since the sensitivity of AFB culture in tuberculous pleurisy remains unsatisfactory, we will use the composite reference standard (CRS) as the diagnostic gold standard for relevant evaluation. Positive AFB smear or culture, radiology consistent with TB, biochemical testing of pleural fluid suggestive of TB, positive immunologic test for TB, positive TB DNA or RNA, pleural pathology supportive of TB, and anti-TB treatment effective comprising the CRS. T (target condition): The target condition will be tuberculous pleurisy. Any type of study that reported on sensitivity, specificity, positive predictive value (PPV), negative predictive value (NPV), and area under the curve (AUC) of NGS for the diagnosis of tuberculous pleurisy and studies in which the original study reported the relevant diagnostic gold standard and where the four values of true positive (TP), false positive (FP), false negative (FN), and true negative (TN) in the diagnostic cross-tabulation can be calculated or directly extracted will be included. Missing information that could be obtained by contacting the corresponding author of the study will also be included.

### Exclusion criteria

Studies with TP, FP, FN, TN values that could not be obtained from the original study and could not be obtained by contacting the corresponding author, case reports, conference abstracts, studies that did not report the full text, reviews, meta-analysis, and studies published in a voice other than Chinese or English will be excluded.

### Information sources

Three English databases (Cochrane Library, Embase and Medline via PubMed) and two Chinese databases (China National Knowledge Infrastructure and Wanfang Database) will be used to search for relevant studies. We will conduct the search of relevant databases in August 2025. References of meta-analysis or reviews on related topics will also be screened.

### Search strategy

Yuyang Ling and Yanqin Shen will cooperate in designing search strategies for relevant databases. We will not restrict the language when searching the individual databases.

A demonstration of the search strategy utilized in PubMed is as follows:

#1 “Tuberculosis, Pleural”[Mesh] OR “Pleural Tuberculos*” OR “Pleural TB” OR “Tuberculoses, Pleural” OR “Pleuris*, Tuberculous” OR “Tuberculous Pleuris*” OR “Pleural Effusion”[Mesh] OR “Effusion*, Pleural” OR “Extrapulmonary tuberculosis” OR “Extra pulmonary tuberculosis”
#2 “Extra-pulmonary tuberculosis” OR “Extra pulmonary tuberculosis” OR “Extrapulmonary tuberculosis” OR EPTB
#3 #1 OR #2
#4 “High-Throughput Nucleotide Sequencing”[Mesh] OR “High Throughput Nucleotide Sequencing” OR “Nucleotide Sequencing, High-Throughput” OR “Sequencing, High-Throughput Nucleotide” OR “Next-Generation Sequencing” OR “Next Generation Sequencing” OR “Sequencing, Next-Generation” OR “High-Throughput DNA Sequencing” OR “DNA Sequencing, High-Throughput” OR “High Throughput DNA Sequencing” OR “Sequencing, High-Throughput DNA” OR “High-Throughput RNA Sequencing” OR “High Throughput RNA Sequencing” OR “RNA Sequencing, High-Throughput” OR “Sequencing, High-Throughput RNA” OR “Massively-Parallel Sequencing” OR “Massively Parallel Sequencing” OR “Sequencing, Massively-Parallel” OR “Deep Sequencing” OR “Sequencing, Deep” OR “Pyrosequencing” OR “Illumina Sequencing” OR “Sequencing, Illumina” OR “Ion Torrent Sequencing” OR “Sequencing, Ion Torrent” OR “Ion Proton Sequencing” OR “Sequencing, Ion Proton” OR “High-Throughput Sequencing” OR “High Throughput Sequencing” OR “Sequencing, High-Throughput”
#5 #3 AND #4

### Literature screening and selection

Literature obtained after searching the relevant databases will first be imported into Endnote (version 9.2) to exclude duplicates and then proceed to the next step of literature screening. Two independent investigators (Yuyang Ling and Yanqin Shen) will evaluate whether the literature meets the requirements of this study by reviewing the title, abstract and full text of the literature in turn, based on the eligibility and exclusion criteria of the study protocol. Once the screening is complete the two individuals will cross-check and any inconsistencies will be discussed with a third person (Guocan Yu) to determine the final results.

### Data extraction

Once the final compliant literature is identified, we will extract the valid information from the literature for statistical analysis. This process will again be done by (Yuyang Ling and Yanqin Shen). First author’s name, publication year, country where the study was conducted, study type, patient selection method (consecutive or non-consecutive), patient number in the study, patient type (pediatric or adult), HIV status, diagnostic gold standard, specimen type (pleural effusion or other), specimen pretreatment method, specimen status (frozen or fresh), type of NGS (targeted or non-targeted amplification), NGS platform (Illumina or others), the cost, where NGS was conducted, and TP, FP, FN, TN values will be the main data to be extracted. Disputes will be handled in the same way as at the screening stage and are decided through discussion with a third party. When the results of two diagnostic gold standards were reported simultaneously in one study, we will distinguish them to be analyzed as two separate studies.

### Quality evaluation

We will assess the quality of the included studies using the Quality Assessment of Diagnostic Accuracy Studies (QUADAS-2) tool [15]. The quality of the studies will be assessed and justified according to the entries in the tool on a case-by-case basis [16], and controversial cases will be handled through third-party resolution.

### Publication bias

The PRISIMA-DTA guideline does not require an assessment of publication bias for meta-analysis of DTA studies because of the flaws in the methodology for evaluating publication bias in DTA studies at present [13].

### Evidence evaluation

We will conduct evidence assessments using the Grading of Recommendations Assessment, Development and Evaluation (GRADE) [17]. In accordance with the guidelines, the level of evidence for each study will be divided into high, moderate, low, and very low.

### Data synthesis and statistical analysis

We will use the four values of TP, FP, FN, and TN to calculate the combined sensitivity, specificity, PPV, NPV, AUC, and their 95% confidence intervals, and then plot the forest plot and receiver operating characteristic curves. We will use the *I*^2^ statistic to assess heterogeneity between included studies, with *I*^2^ = 0 suggesting no heterogeneity between studies and *I*^2^ > 50% suggesting significant heterogeneity between studies [18]. We will conduct meta-regression analysis, subgroup analysis and sensitivity analysis to explore possible sources of heterogeneity when significant heterogeneity exists. We will conduct meta-regression analysis and subgroup analysis based on the following parameters: study type, patient selection method, patient type, HIV status, specimen type, specimen pretreatment method, specimen status, type of NGS, NGS platform. Sensitivity analysis will be conducted by removing a study and providing the results of the analysis with and without this study to assess whether the study is a high-risk study. The sensitivity analysis will assess the reliability and robustness of the combined results. When there are more than 4 eligible studies, we will use Stata (version 15.0; Stata Corp., College Station, TX, the USA) with *midas* module to conduct the relevant meta-analysis and plot the relevant forest plots and curves using bivariate random-effects models. When there are less than 4 eligible studies, we will use Meta-DiSc (version 1.4) to perform the associated meta-analysis and plot the associated forest plots and curves.

## Supporting information

S1 File Preferred Reporting Items for Systematic review and Meta-Analysis Protocols (PRISMA-P) checklist. (DOCX)

S2 File Search strategies for Chinese and English databases. (DOCX)
