# Supplementary material for: Accuracy of next-generation sequencing for rapid diagnosis of tuberculous pleurisy: A protocol of systematic review and meta-analysis
Source: PLoS One. 2025 Feb 25;20(2):e0319175. doi: 10.1371/journal.pone.0319175 (PMC11856282; doi:10.1371/journal.pone.0319175)
Supplement: S2 File — (DOCX) [file pone.0319175.s002.docx]

Pubmed, Embase and Cochrane

#1 "Tuberculosis, Pleural"[Mesh] OR “Pleural Tuberculos*” OR “Pleural TB” OR “Tuberculoses, Pleural” OR “Pleuris*, Tuberculous” OR “Tuberculous Pleuris*” OR "Pleural Effusion"[Mesh] OR “Effusion*, Pleural” OR “Extrapulmonary tuberculosis” OR “Extra pulmonary tuberculosis”

#2 “Extra-pulmonary tuberculosis” OR “Extra pulmonary tuberculosis” OR “Extrapulmonary tuberculosis” OR EPTB

#3 #1 OR #2

#4 “High-Throughput Nucleotide Sequencing”[Mesh] OR “High Throughput Nucleotide Sequencing” OR “Nucleotide Sequencing, High-Throughput” OR “Sequencing, High-Throughput Nucleotide” OR “Next-Generation Sequencing” OR “Next Generation Sequencing” OR “Sequencing, Next-Generation” OR “High-Throughput DNA Sequencing” OR “DNA Sequencing, High-Throughput” OR “High Throughput DNA Sequencing” OR “Sequencing, High-Throughput DNA” OR “High-Throughput RNA Sequencing” OR “High Throughput RNA Sequencing” OR “RNA Sequencing, High-Throughput” OR “Sequencing, High-Throughput RNA” OR “Massively-Parallel Sequencing” OR “Massively Parallel Sequencing” OR “Sequencing, Massively-Parallel” OR “Deep Sequencing” OR “Sequencing, Deep” OR “Pyrosequencing” OR “Illumina Sequencing” OR “Sequencing, Illumina” OR “Ion Torrent Sequencing” OR “Sequencing, Ion Torrent” OR “Ion Proton Sequencing” OR “Sequencing, Ion Proton” OR “High-Throughput Sequencing” OR “High Throughput Sequencing” OR “Sequencing, High-Throughput”

#5 #3 AND #4

CNKI AND Wanfang

#1 结核性胸膜炎 OR 胸腔积液 OR 胸膜结核 OR胸水 OR 肺外结核

#2 下一代测序 OR 高通量测序测序 OR 核酸测序 OR DNA测序 OR RNA 测序 OR 基因组测序 OR NGS

#3 #1 AND #2
